# Supplementary material for: Sex Bias in Frailty Screening: A Cross-Sectional Analysis of PRISMA-7 and the Clinical Frailty Scale in Primary Care
Source: Diagnostics (Basel). 2025 Apr 2;15(7):915. doi: 10.3390/diagnostics15070915 (PMC11989021; doi:10.3390/diagnostics15070915)
Supplement: Supplementary file 1 [file diagnostics-15-00915-s001.zip › diagnostics-3478536-supplementary.pdf]

Supplementary File S1: PRISMA-7 and Clinical Frailty Scale (CFS) Items

PRISMA-7 Items

The PRISMA-7 (Program of Research to Integrate the Services for the Maintenance of Autonomy) is a 7-item questionnaire used to screen for frailty in older adults. Each item is scored 1 (yes) or 0 (no), with a score ≥3 indicating frailty. The items are:

1. 1. Are you older than 85 years?
2. 2. Are you male?
3. 3. In general, do you have any health problems that require you to limit your activities?
4. 4. Do you need someone to help you on a regular basis?
5. 5. In general, do you have any health problems that require you to stay at home?
6. 6. In case of need, can you count on someone close to you? (reverse scored)
7. 7. Do you regularly use a cane, a walker, or a wheelchair to get about?

Clinical Frailty Scale (CFS) Descriptions

The Clinical Frailty Scale (CFS) is a clinician-rated tool that classifies patients from 1 (very fit) to 9 (terminally ill) based on their level of fitness and function. It provides a global clinical judgment of frailty. The levels are:

8. 1. Very Fit – Robust, active, energetic, and motivated. These people commonly exercise regularly and are among the fittest for their age.
9. 2. Well – No active disease symptoms but less fit than category 1. Often, they exercise or are very active occasionally, e.g., seasonally.
10. 3. Managing Well – Medical problems are well controlled, but not regularly active beyond routine walking.
11. 4. Vulnerable – While not dependent on others for daily help, symptoms limit activities. A common complaint is being 'slowed up' or being tired during the day.
12. 5. Mildly Frail – With limited dependence on others for instrumental activities of daily living.
13. 6. Moderately Frail – Help is needed with both instrumental and basic activities of daily living.
14. 7. Severely Frail – Completely dependent for personal care, from whatever cause (physical or cognitive). Even so, they seem stable and not at high risk of dying.
15. 8. Very Severely Frail – Completely dependent, approaching the end of life. Typically, they could not recover even from a minor illness.
16. 9. Terminally Ill – Approaching the end of life. This category applies to people with a life expectancy <6 months, who are not otherwise evidently frail.
